# Differences in structural brain morphometry between musicians and non-musicians

**DOI:** 10.3389/fnhum.2025.1638813

**Published:** 2025-10-30

**Authors:** Ying Liu, Qilin Huang, Thomas Hosseini, Jiancheng Hou

**Affiliations:** ^1^Academy of Music, Southwest University, Chongqing, China; ^2^Mental Health Research Institute of Chinese Music, Southwest University, Chongqing, China; ^3^Department of Radiology, School of Medicine and Public Health, University of Wisconsin-Madison, Madison, WI, United States; ^4^Research Center for Cross-Straits Cultural Development, Fujian Normal University, Fuzhou, Fujian, China

**Keywords:** structural brain morphometry, gender, musical training, musicians, non-musicians

## Abstract

**Objective:**

Although music has been shown to affect brain function, the structural characteristics of the brain in musicians compared to non-musicians are often overlooked. This limited attention restricts the practical use of music’s emotional, cognitive, and motor functions. The current study aimed to investigate structural differences in the brains of musicians compared to non-musicians in order to better understand the neuroanatomical basis of musical training.

**Methods:**

Sixteen musicians and seventeen age-matched non-musicians underwent a brain structural neuroimaging scan. Group differences in structural morphometry were assessed.

**Results:**

Significant differences were found in cortical thickness, fractal dimensionality, gyrification, and sulcal depth measures. Compared to non-musicians, musicians showed greater cortical thickness in the left superior frontal gyrus and right central parietal region, and showed structural advantages in fractal dimensionality and sulcal depth in the left fusiform gyrus and right central region. In contrast, non-musicians showed greater gyrification in the bilateral insula, right superior parietal lobule, and right supramarginal gyrus. Notably, significant interactive effects were observed between gender and cortical thickness, fractal dimensionality, gyrification, and sulcal depth in regions of the limbic system, including the hippocampus, cingulate gyrus, insula, fusiform gyrus, and precuneus.

**Conclusion:**

Structural differences in the frontal cortex, limbic system, and sensorimotor areas between musicians and non-musicians highlight the changes in brain structure associated with musical training. These findings provide insight into the underlying mechanisms of music-related brain function and may provide guidance for future applications of music to improve mental health and neuroplasticity.

## 1 Introduction

The brain is not a static organ; rather, it changes dynamically across an individual’s lifespan. Throughout human development, the brain adapts in response to various experiences. Music, as one of the most important experiences of emotion regulation, not only enhances emotional experience, cognitive processing, and social interaction, but also affects the morphological and functional properties of internal cranial nerves. Long term exposure to music—whether it be through listening, active training, or passive exposure—can induce stable morphological changes in the brain, continuing to shape one’s cognitive processing, emotional expression, and behavioral responses. These music-induced changes in plasticity, often referred to as music-based interventions (Sihvonen et al., 2017), have become a growing focus in neurological rehabilitation in recent years.

Traditional studies on music and the brain have mainly focused on how musical training affects functional neural activity. One key finding from these studies is that the dopamine reward system—comprised of the hippocampus, hypothalamus, ventral tegmental area, and nucleus accumbens—is activated during music-evoked emotion (Koelsch, 2010; Sachs et al., 2015). This reward system also interacts with functional structures involved in auditory perception (as a predictive process) to enhance an individual’s hedonic experience (Belfi et al., 2019), and contributes to emotion regulation, empathic feelings, and prosocial behavior (Ferreri et al., 2019). Even in extremely preterm infants, music exposure has been shown to enhance the structural maturation of emotion-related neural pathways—such as in the external capsule, claustrum, extreme capsule, and uncinate fasciculus—and to increase amygdala volume when compared to infants receiving the standard-of-care (Sa de Almeida et al., 2020).

Musicians also demonstrate superior integration of motor patterns and sensory processing across somatosensory and auditory domains. Structural and functional advantages have been observed in areas such as the posterior-superior cerebellar hemisphere, the dominant primary sensorimotor cortex, the left Heschl’s gyrus (Gebel et al., 2013), and fractional anisotropy (Halwani et al., 2011). When comparing brain network activity during cello playing and singing, overlapping activation was observed in the intraparietal sulcus (IPS) and supramarginal gyrus (SMG) when participants were asked to either compensate for or ignore introduced pitch perturbations, and in the posterior superior temporal gyrus (pSTG) and dorsal pre-motor cortex (dPMC) during the same task. Differences between singing and playing were most prominent in the primary motor cortex (M1), centered on the relevant motor effectors (e.g., hand, larynx) (Segado et al., 2021).

However, the human brain also functions during resting states, not just during active tasks. A well-regulated resting state supports more effective brain function during task performance, and music is an important stimulus that can influence brain structure even at rest. Just as mindfulness meditation influences brain cortical structure, musical training can also lead to non-task related structural changes—such as alterations in cortical thickness, fractal dimensionality, gyrification, and sulcal depth—which may enhance cognitive processing and regulate neural activity during resting or non-task states.

Most research up to this point has focused on differences in cortical thickness and surface area in sensorimotor regions when comparing brain surface morphometry in musicians and non-musicians. One study identified 17 regions—9 cerebellar and 8 sensorimotor—that differed between musicians and non-musicians, as well as between early-learning and late-learning musicians (Shenker et al., 2023). Moreover, musicianship was found to be correlated with greater cortical thickness and gray matter volumes in the frontal and temporal regions, and musicians also showed more localized structural whole-brain covariance compared to non-musicians (Bermudez et al., 2009). These findings support the commonly accepted view that musical activity requires sophisticated dynamic interplay between multisensory and motor behaviors, sub-served by the auditory, visual, tactile, and motor systems of the brain in particular (Moller et al., 2021).

Cortical thickness examines the distance between the top of the brain and the white matter boundary in the neocortex, while also examining gray matter morphology (Hirakawa et al., 2016; Seiger et al., 2018). However, this measure is limited to cortical areas, and does not capture information from non-cortical areas of the brain (Bermudez et al., 2009). Therefore, additional measures—such as fractal dimensionality, gyrification, and sulcal depth—can complement cortical thickness to make up for its limitations. Fractal dimensionality examines structural complexity beyond the capabilities of cortical thickness (Di Ieva et al., 2014, 2015; Madan and Kensinger, 2017; Yotter et al., 2011b), and has been shown to be more sensitive to structural variability (Chen et al., 2020; Madan and Kensinger, 2017; Zhang et al., 2008). Gyrification reflects the amount of local cortical folding, thus serving as an indicator for the integrality between cortical and subcortical circuits (Li et al., 2021). Sulcal depth, measured as the Euclidean distance between the cortex and outer surface (Li et al., 2021; Yun et al., 2013), captures changes in gray and white matter (Im et al., 2008; Jin et al., 2018; Kim et al., 2008; Kochunov et al., 2008). Because sulcal depth is not limited to gray matter alone, it is sensitive to the complex folding patters of the cortical surface, and serves as a relatively new way to observe the cerebral cortex (Jin et al., 2018).

In order to investigate the effects of musical training on brain structure, the current study examined cortical thickness, fractal dimensionality, gyrification, and sulcal depth in a group of age-matched musicians and non-musicians. Additionally, we also examined the gender effect on brain surface morphometry between musicians and non-musicians, as it is known that gender can influence neural processing during music perception. For example, an event-related potentials (ERP) study found that females display early right anterior negativity (ERAN) and mismatch negativity (MMN) in both hemispheres during music syntactic processing, whereas males exhibit right hemispheric dominance (Koelsch et al., 2003). A neuroimaging study also found that, compared to females, males generally rely on the left lateralized hemisphere (e.g., the anterior and posterior perisylvian areas and cerebellum) during music pitch processing (Gaab et al., 2003). Therefore, the goal of this study was to identify key structural differences between musicians and non-musicians, and to examine how gender may influence these differences. By doing so, we hope to contribute to the growing body of work on music-induced neuroplasticity and its implications for cognitive and emotional functioning.

## 2 Methods

### 2.1 Data source

Structural T1-weighted images (NIFTI format) were obtained from a public dataset via OpenNeuro with accession number ds003146.^1^ There were sixteen musicians (age range: 20–42 years old, mean age: 28 ± 7 years old; 8 males and 8 females) who had at least 3 years of formal musical training or studies experiences (either singing or playing instruments) who were also currently involved in daily musical activities. Fourteen musicians practiced multiple instruments (such as piano, keyboard, guitar, saxophone, bass etc.), although there was one or two main instruments practiced. Among them, four singing musicians also had instrumental practice, although they mainly had singing practice (classified as singing musicians). In addition, two musicians only practiced the piano. Their mean practice years were 19 ± 12.264 years, the age of music practicing onset was 11.188 ± 6.473 years, and weekly practice time was 12.688 ± 8.404 hours. The participants’ detailed information can be seen in the S1 Table of https://doi.org/10.1371/journal.pone.0222796. Seventeen age-matched non-musicians (age range: 20–45 years old, mean age: 27 ± 6 years old; 8 males and 9 females) did not receive extra-curricular music instruction beyond a mandatory school music course. All participants were Spanish-speaking, right-handed, and had normal hearing. They gave their written informed consent before the MRI scanning session. The research protocol was approved by the Ethics Committee of the Institute of Neurobiology at the Universidad Nacional Autónoma de México and was conducted in accordance with the international standards of the Declaration of Helsinki of 1964 (Angulo-Perkins and Concha, 2019).

### 2.2 MRI data acquisition

Images were acquired on a 3 T Discovery MR750 scanner (General Electric, Waukesha, Wisconsin) with a 32-channel coil. The parameters were: TR = 2,300 ms, TE = 3 ms, feld of view = 256 × 256 mm^2^, voxel size = 1 × 1 × 1 mm^3^.

### 2.3 Cortical surface preprocessing

Image preprocessing was performed with the Computational Anatomy Toolbox (CAT12, http://www.neuro.uni-jena.de/cat/), a software plugin for Statistical Parametric Mapping (SPM12, https://www.fil.ion.ucl.ac.uk/spm/software/spm12/) within MATLAB. CAT12 is not only more precise and accurate compared to earlier voxel-based morphometry (VBM) plug-ins (Farokhian et al., 2017; Yuksel et al., 2018), but is also fully automated for surface-based analysis (Zhuang et al., 2017). The preprocessing steps in CAT12 consisted of bias-field correction, skull-stripping, alignment to the Montreal Neurological Institute (MNI) structural template (to classify gray matter (GM), white matter (WM) and cerebrospinal fluid (CSF), and spatial normalization [with the Diffeomorphic Anatomical Registration Through Exponentiated Lie Algebra (DARTEL) registration 1.5 mm)] (Kurth et al., 2015; Yuksel et al., 2018; Zhuang et al., 2017). After these initial preprocessing steps, a spherical harmonic approach (Yotter et al., 2011a) was used to reparametrize the brain surface mesh in order to reduce brain area distortions (Yotter et al., 2011c) and repair topological defects (Chen et al., 2020; Yotter et al., 2011a,c).

Cortical thickness was preprocessed based on the established CAT12 workflow (Dahnke et al., 2013). This algorithm uses tissue segmentation to evaluate WM distance and projects the local maxima to the GM voxels. Values at the outer GM boundary within the WM distance map are then projected back to the inner GM boundary to generate GM thickness (Li et al., 2021). A central surface was then generated between the GM thickness and WM distance (Li et al., 2021). Spatial normalization was applied with the DARTEL registration (Li et al., 2021), and spatial smoothing was performed using a 15 mm full-width at half maximum (FWHM) Gaussian kernel.

Fractal dimensionality, which examines cortical complexity, was derived from spherical harmonic reconstructions (Li et al., 2021; Yotter et al., 2011a). This technique calculates the slope between a logarithmic plot of surface area and the maximum value, representing the bandwidth of frequencies used to reconstruct the cortical surface shape (Li et al., 2021; Yotter et al., 2011b). Spatial smoothing for the fractal dimensionality was performed using a 20 mm FWHM Gaussian kernel.

Gyrification, considered an indicator of cortical folding, was calculated using absolute mean curvature, an extrinsic surface measure that captures changes in the normal direction of the brain surface (Li et al., 2021; Luders et al., 2006). Spatial smoothing was once again performed with a 20 mm FWHM Gaussian kernel.

Sulcal depth was calculated as the Euclidean distance between the central surface and its convex hull, and the resulting values were then transformed using the sqrt function (Li et al., 2021). Spatial smoothing for sulcal depth was also performed using a 20 mm FWHM Gaussian kernel.

### 2.4 Statistical analysis

The current study used a two-factor between-subjects design with music group (musicians vs. non-musicians) and gender (males vs. females) as independent variables. Two-way (music group × gender) ANCOVAs were conducted to examine differences between the groups on the main study variables, with age and intracranial volumes considered as covariates. All morphometric analyses were performed using CAT12 and were analyzed via a non-parametric permutation technique. Threshold-Free Cluster Enhancement (TFCE) was used in permutation testing with 5,000 permutations (Smith and Nichols, 2009). TFCE *p* < 0.05 was used for multiple comparison correction. The brain regions with cluster size of at least 100 vertices (cluster size × percentage covered in the specific region produced by CAT12) were reported. The Desikan–Killiany atlas (DK40) (Desikan et al., 2006) was used to label the cortical regions and the results were visualized using CAT12.

## 3 Results

### 3.1 Age

As expected, the two-way ANOVA revealed no significant age differences among the four subgroups (group: *F _(1,33)_ =* 1.123, *η*^2^ = 0.037, *p* = 0.298; gender: *F _(1,33)_ =* 0.230, *η*^2^ = 0.008, *p* = 0.635; group x gender: *F _(1,33)_ =* 1.034, *η*^2^ = 0.034, *p* = 0.318), indicating that participants were successfully matched.

### 3.2 Structural morphometry differences

#### 3.2.1 Cortical thickness

**Figure 1:**
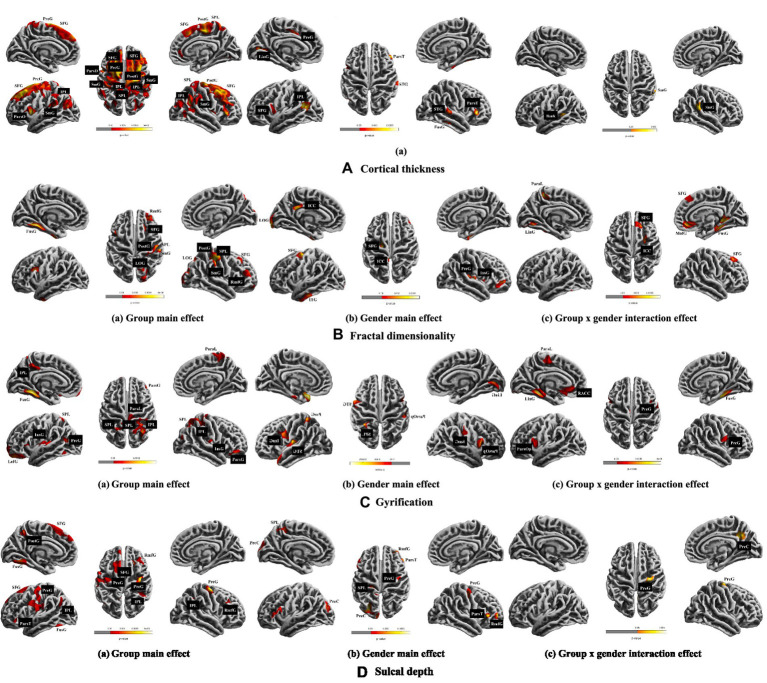
Results of ANCOVAs (with age and cranium volume as covariates) for all main variables. The multiple comparison correction was used with non-parametric permutations (*n* = 5,000) and threshold-free cluster enhancement (TFCE) correction *p* < 0.05, cluster size > 100. The brain left/right hemisphere in the figure is also the actual brain left/right hemisphere. **A**, **B**, **C**, and **D** shows the cortical thickness, fractal dimensionality, gyrification, and sulcal depth, respectively. SFG, superior frontal gyrus; SPL, superior parietal lobule; IPL, inferior parietal lobule; ParaL, paracentral lobule; ParsO, parsopercularis; ParsT, Parstriangularis; ParsG, parsopercularis gyrus; SmG, supramarginal gyrus; PreG, precentral gyrus; PostG, postcentral gyrus; LinG, lingual gyrus; STG, superior temporal gyrus; ITG, inferior temporal gyrus; Bank, bankssts gyrus; RmfG, rostral middle frontal gyrus; LOG, lateral occipital gyrus; ICC, isthmus cingulate cortex; InsG, insula gyrus; FusG, fusiform gyrus; LofG, lateral orbitofrontal gyrus; PreC, precuneus; RACC, rostral anterior cingulate cortex.

(1) The main effect of musical training was observed in the left pars opercularis and precentral gyri, postcentral gyrus, superior parietal lobule, and in the superior frontal, supramarginal gyri, and inferior parietal lobule in both hemispheres. (2) Gender had a main effect on the left lingual, precentral, superior frontal gyri, and inferior parietal lobule as well as the right fusiform, superior temporal, and pars triangularis gyri. (3) Musical training and gender had a significant interaction effect in the left banks of the superior temporal sulcus (bankssts) and right supramarginal gyrus (see Table 1 and Figure 1A).

**Table 1:** Results of ANCOVAs (with age and cranium volume as covariates) for all main variables.

Measures	Main and interaction effect	Regions	Cluster size	*p* value
Cortical thickness	Group	*Left hemisphere*		
		Superior frontal gyrus	1,436	0.000
		Inferior parietal lobule	460	0.002
		Parsopercularis	210	0.000
		Supramarginal gyrus	473	0.001
		Precentral gyrus	105	0.002
		*Right hemisphere*		
		Superior frontal gyrus	1,094	0.000
		Inferior parietal lobule	761	0.000
		Superior parietal lobule	392	0.003
		Supramarginal gyrus	416	0.001
		Postcentral gyrus	181	0.000
	Gender	*Left hemisphere*		
		Inferior parietal lobule	490	0.000
		Lingual gyrus	115	0.001
		Precentral gyrus	100	0.007
		Superior frontal gyrus	105	0.007
		*Right hemisphere*		
		Fusiform	123	0.003
		Superior temporal gyrus	113	0.006
		Parstriangularis	121	0.000
	Group x Gender	*Left hemisphere*		
		Bankssts gyrus	100	0.006
		*Right hemisphere*		
		Supramarginal gyrus	221	0.007
Fractal dimensionality	Group	*Left hemisphere*		
		Fusiform	224	0.000
		*Right hemisphere*		
		Postcentral gyrus	484	0.000
		Rostral middle frontal gyrus	337	0.003
		Lateral occipital gyrus	156	0.001
		Superior frontal gyrus	129	0.002
		Superior parietal lobule	119	0.002
		Supramarginal gyrus	119	0.000
	Gender	*Left hemisphere*		
		Inferior temporal gyrus	132	0.004
		Superior frontal gyrus	107	0.001
		Lateral occipital gyrus	100	0.001
		Isthmus cingulate cortex	105	0.001
		*Right hemisphere*		
		Insula gyrus	147	0.000
		Precentral gyrus	132	0.009
		Fusiform	102	0.005
	Group x Gender	*Left hemisphere*		
		Lingual gyrus	162	0.005
		Paracentral lobule	134	0.000
		*Right hemisphere*		
		Fusiform	211	0.001
		Isthmus cingulate cortex	166	0.000
		Medial orbitofrontal gyrus	155	0.001
		Superior frontal gyrus	173	0.003
Gyrification	Group	*Left hemisphere*		
		Fusiform	350	0.000
		Insula gyrus	332	0.003
		Lateral orbitofrontal gyrus	273	0.001
		Precuneus	175	0.004
		Superior parietal lobule	136	0.015
		Inferior parietal lobule	117	0.002
		*Right hemisphere*		
		Superior parietal lobule	413	0.002
		Paracentral lobule	177	0.009
		Inferior parietal lobule	268	0.001
		Insula gyrus	133	0.011
		Parsorbitalis gyrus	146	0.001
	Gender	*Left hemisphere*		
		Supramarginal gyrus	119	0.007
		Parsopercularis gyrus	162	0.003
		Lingual gyrus	105	0.005
		*Right hemisphere*		
		Superior temporal gyrus	292	0.000
		Insula gyrus	333	0.001
		Precentral gyrus	253	0.004
		Superior parietal lobule	123	0.001
	Group x Gender	*Left hemisphere*		
		Parahippocampal gyrus	261	0.000
		Parsopercularis gyrus	155	0.004
		Rostral anterior cingulate cortex	101	0.003
		Lingual gyrus	101	0.004
		Paracentral lobule	133	0.004
		*Right hemisphere*		
		Fusiform	255	0.000
		Precentral gyrus	126	0.004
Sulcal depth	Group	*Left hemisphere*		
		Postcentral gyrus	467	0.005
		Superior frontal gyrus	494	0.008
		Fusiform	334	0.002
		Precentral gyrus	317	0.004
		Inferior parietal lobule	140	0.010
		Precuneus	127	0.002
		Parstriangularis gyrus	101	0.018
		*Right hemisphere*		
		Precentral gyrus	237	0.000
		Rostral middle frontal gyrus	164	0.008
		Inferior parietal lobule	110	0.017
	Gender	*Left hemisphere*		
		Superior parietal lobule	243	0.000
		Precuneus	130	0.010
		*Right hemisphere*		
		Precentral gyrus	180	0.006
		Parstriangularis gyrus	111	0.001
		Rostral middle frontal gyrus	101	0.000
	Group x Gender	*Right hemisphere*		
		Precuneus	215	0.006
		Precentral gyrus	112	0.006

The multiple comparison correction was used with non-parametric permutations (*n* = 5,000) and threshold-free cluster enhancement (TFCE) correction *p* < 0.05, cluster size > 100.

*Post hoc* analysis indicated the following: (1) Male musicians exhibited greater cortical thickness in the left superior frontal, lingual, precentral, and inferior parietal lobule, as well as in the right superior temporal and pars triangularis gyri, but reduced cortical thickness in the right fusiform gyrus compared to female musicians. (2) Male non-musicians showed greater cortical thickness in the left lingual gyrus compared to female non-musicians. (3) Male musicians had greater cortical thickness compared to male non-musicians in the left superior frontal, supramarginal, bankssts gyri, and inferior parietal lobule, as well as in the right superior frontal, superior and inferior parietal lobules (4) Female musicians exhibited increased cortical thickness compared to female non-musicians in the left superior frontal, supramarginal, pars opercularis gyri, inferior parietal lobule, and in the right superior frontal, postcentral gyri, and superior and inferior parietal lobules (see Table 2).

**Table 2:** *Post hoc* analysis (with age and cranium volume as covariates) for all main variables.

Measures	Group comparisons	Regions	Cluster size	*p* value
Cortical thickness	Male musicians > female musicians	*Left hemisphere*		
		Inferior parietal lobule	497	0.000
		Lingual gyrus	125	0.002
		Superior frontal gyrus	293	0.009
		Precentral gyrus	193	0.002
		*Right hemisphere*		
		Superior temporal gyrus	170	0.001
		Parstriangularis gyrus	212	0.004
	Male musicians < female musicians	*Right hemisphere*		
		Fusiform	202	0.002
	Male nonmusicians > female nonmusicians	*Left hemisphere*		
		Lingual gyrus	143	0.002
	Male musicians > male nonmusicians	*Left hemisphere*		
		Superior frontal gyrus	1,265	0.000
		Inferior parietal lobule	447	0.002
		Supramarginal gyrus	212	0.003
		bankssts	115	
		*Right hemisphere*		
		Superior frontal gyrus	1,061	0.001
		Inferior parietal lobule	334	0.001
		Superior parietal lobule	164	0.014
	Female musicians > female nonmusicians	*Left hemisphere*		
		Superior frontal gyrus	1,314	0.000
		Supramarginal gyrus	657	0.001
		Inferior parietal lobule	391	0.000
		Parsopercularis gyrus	246	0.000
		*Right hemisphere*		
		Superior frontal gyrus	831	0.000
		Postcentral gyrus	149	0.005
		Inferior parietal lobule	112	0.018
		Superior parietal lobule	200	0.001
Fractal dimensionality	Male musicians > female musicians	*Left hemisphere*		
		Inferior temporal gyrus	232	0.000
		Superior frontal gyrus	140	0.003
		*Right hemisphere*		
		Precentral gyrus	222	0.001
		Isthmus cingulate cortex	157	0.001
		Medial orbitofrontal gyrus	153	0.012
	Male musicians < female musicians	*Left hemisphere*		
		Isthmus cingulate cortex	134	0.000
		*Right hemisphere*		
		Fusiform	291	0.000
		superiorfrontal	313	0.000
	Male nonmusicians > female nonmusicians	*Left hemisphere*		
		Superior frontal gyrus	299	0.001
		Paracentral lobule	158	0.000
		Inferior temporal gyrus	124	0.000
		*Right hemisphere*		
		Insula	356	0.003
		Fusiform	134	0.005
		Superior frontal gyrus	113	0.008
	Male nonmusicians < female nonmusicians	*Left hemisphere*		
		Lingual gyrus	230	0.001
	Male musicians > male nonmusicians	*Left hemisphere*		
		Lingual gyrus	180	0.000
		*Right hemisphere*		
		Postcentral gyrus	330	0.000
		Rostral middle frontal gyrus	278	0.007
	Male musicians < male nonmusicians	*Right hemisphere*		
		Superior parietal lobule	195	0.007
		Superior frontal gyrus	191	0.008
		Supramarginal gyrus	145	0.001
	Female musicians > female nonmusicians	*Left hemisphere*		
		Paracentral lobule	173	0.000
		Fusiform	171	0.001
		*Right hemisphere*		
		Postcentral gyrus	350	0.001
		Superior frontal gyrus	190	0.006
	Female musicians < female nonmusicians	*Right hemisphere*		
		Superior parietal lobule	108	0.000
		Rostral middle frontal gyurs	172	0.002
Gyrification	Male musicians > female musicians	*Left hemisphere*		
		Parahippocampal gyrus	234	0.009
		*Right hemisphere*		
		Superior temporal gyrus	398	0.004
		Fusiform	310	0.000
		Superior parietal lobule	227	0.003
		Precentral gyrus	228	0.002
	Male musicians < female musicians	*Right hemisphere*		
		Insula gyrus	142	0.010
	Male nonmusicians > female nonmusicians	*Left hemisphere*		
		Lingual gyrus	173	0.004
		*Right hemisphere*		
		Precentral gyrus	262	0.002
		Superior temporal gyrus	133	0.002
	Male nonmusicians < female nonmusicians	*Left hemisphere*		
		Paracentral lobule	372	0.000
		Parsopercularis gyrus	214	0.003
		*Right hemisphere*		
		Insula gyrus	297	0.002
	Male musicians < male nonmusicians	*Left hemisphere*		
		Precuneus	130	0.000
		*Right hemisphere*		
		Superior parietal lobule	194	0.000
		Insula gyrus	101	0.000
		Paracentral lobule	165	0.000
	Female musicians < female nonmusicians	*Left hemisphere*		
		Insula gyrus	405	0.002
		Fusiform gyrus	295	0.000
		Precuneus	257	0.004
		Lateral orbitofrontal gyrus	117	0.002
		Inferior parietal lobule	163	0.007
		Superior parietal lobule	110	0.013
		*Right hemisphere*		
		Paracentral lobule	472	0.000
		Fusiform	251	0.000
Sulcal depth	Male musicians > female musicians	*Left hemisphere*		
		Superior parietal lobule	218	0.009
	Male musicians < female musicians	*Left hemisphere*		
		Precuneus	130	0.002
		*Right hemisphere*		
		Precentral gyrus	195	0.003
		Rostral middle frontal gyrus	162	0.003
		precuneus	162	0.003
	Male nonmusicians < female nonmusicians	*Left hemisphere*		
		Precentral gyrus	205	0.006
		Superior parietal lobule	100	0.005
		*Right hemisphere*		
		Parstriangularis gyrus	135	0.002
		Rostral middle frontal gyrus	110	0.000
	Male musicians > male nonmusicians	*Left hemisphere*		
		Fusiform	394	0.003
	Male musicians < male nonmusicians	*Left hemisphere*		
		Postcentral gyrus	540	0.004
		Precuneus	176	0.008
		Superior frontal gyrus	220	0.021
		Precentral gyrus	238	0.012
		*Right hemisphere*		
		Precentral gyrus	344	0.000
	Female musicians > female nonmusicians	*Right hemisphere*		
		Precuneus	539	0.000
	Female musicians < female nonmusicians	*Left hemisphere*		
		Superior frontal gyrus	447	0.001
		Precentral gyrus	205	0.002
		Inferior parietal lobule	199	0.001
		*Right hemisphere*		
		Rostral middle frontal gyrus	207	0.004

The multiple comparison correction was used with non-parametric permutations (*n* = 5,000) and threshold-free cluster enhancement (TFCE) correction *p* < 0.05, cluster size >100.

#### 3.2.2 Fractal dimensionality

(1) Musical training had a main effect on the left fusiform gyrus and the right postcentral, rostral middle frontal, superior frontal, lateral occipital, supramarginal gyri, and superior parietal lobule. (2) Gender had a main effect on the left inferior temporal, superior frontal, lateral occipital and isthmus cingulate gyri, as well as on the right insula, precentral, and fusiform gyri. (3) Interaction effects between musical training and gender were seen in the left lingual gyrus and paracentral lobule, and in the right fusiform, isthmus cingulate, medial orbitofrontal, and superior frontal gyri (see Table 1 and Figure 1B).

*Post hoc* analysis showed that: (1) Male musicians had greater fractal dimensionality in the left superior frontal and inferior temporal gyri, and in the right precentral, medial orbitofrontal gyri, and isthmus cingulate cortex, but reduced fractal dimensionality in the left isthmus cingulate cortex and right superior frontal and fusiform gyri, compared to female musicians. (2) Male non-musicians had higher fractal dimensionality in the left superior frontal and inferior temporal gyri and paracentral lobule, as well as in the right insula, fusiform, and superior frontal gyri, but lower fractal dimensionality in the left lingual gyrus, than female nonmusicians. (3) Male musicians had increased fractal dimensionality compared to male non-musicians in the left lingual gyrus and right postcentral and rostral middle frontal gyri, but had reduced values in the right superior frontal, supramarginal gyri, and superior parietal lobule. (4) Female musicians had increased fractal dimensionality compared to female non-musicians in the left fusiform gyrus and paracentral lobule, and in the right postcentral and superior frontal gyri, but reduced values in the right rostral middle frontal gyrus and superior parietal lobule (see Table 2).

#### 3.2.3 Gyrification

(1) Musical training had a main effect on the left fusiform, lateral orbitofrontal, and precuneus gyri, and on the right pars orbitalis gyrus and paracentral lobule, as well as on the insula gyrus and superior and inferior parietal lobules in both hemispheres. (2) Gender had a main effect on the left supramarginal, pars opercularis and lingual gyri, and on the right superior temporal, insula, precentral gyri and superior parietal lobule. (3) Interaction effects between musical training and gender were observed in the left parahippocampus, pars opercularis, rostral anterior cingulate, lingual gyri, and paracentral lobule, and in the right fusiform and precentral gyri (see Table 1 and Figure 1C).

*Post hoc* analysis showed that: (1) Male musicians had increased gyrification in the left parahippocampus and in the right superior temporal, fusiform, and precentral gyri, and superior parietal lobule, but had reduced gyrification in the right insula compared to female musicians. (2) Male non-musicians exhibited greater gyrification in the left lingual gyrus and right precentral and superior temporal gyri, but had reduced gyrification in the left pars opercularis, paracentral lobule, and right insula compared to female non-musicians. (3) Male musicians had reduced gyrification compared to male nonmusicians in the left precuneus and in the right insula, superior parietal, and paracentral lobules. (4) Female musicians showed reduced gyrification compared to female non-musicians in the left insula, fusiform, precuneus, lateral orbitofrontal gyri, and superior and inferior parietal lobules, as well as in the right fusiform gyrus and paracentral lobule (see Table 2).

#### 3.2.4 Sulcal depth

(1) Musical training had a main effect on the left postcentral, superior frontal, fusiform, precuneus, and pars triangularis gyri, on the right rostral middle frontal gyrus, and on the precentral gyrus and inferior parietal lobule in both hemispheres. (2) Gender had a main effect on the left precuneus and superior parietal lobule, and on the right precentral, pars triangularis, and rostral middle frontal gyri. (3) Musical training and gender had interaction effects in the right precuneus and precentral gyri (see Table 1 and Figure 1D).

Post hoc analysis showed that: (1) Male musicians had greater sulcal depth in the left superior parietal lobule, but had reduced sulcal depth in the left precuneus and in the right precentral, rostral middle frontal, and precuneus compared to female musicians. (2) Male non-musicians exhibited reduced sulcal depth compared female non-musicians in the left precentral gyrus and superior parietal lobule, as well as in the right pars triangularis and rostral middle frontal gyri. (3) Male musicians had greater sulcal depth compared to male non-musicians in the left fusiform gyrus, but reduced sulcal depth in the left postcentral, precentral, precuneus, and superior frontal gyri, as well as in the right precentral gyrus. (4) Female musicians showed increased sulcal depth in the right precuneus, but decreased sulcal depth in the left superior frontal and precentral gyri, inferior parietal lobule, and in the right rostral middle frontal gyrus compared to female non-musicians (see Table 2).

## 4 Discussion

In the current study, we investigated differences in cortical thickness, fractal dimensionality, gyrification, and sulcal depth between musicians and non-musicians. In addition to comparing the effects of musical training on brain structural development, we also analyzed how these structural indicators vary across specific brain regions in conjunction with gender characteristics.

### 4.1 Cortical thickness—musicians versus non-musicians

Cortical thickness in the frontal regions of the brain serves as important evidence for the structural impact of musical training. In the current study, musicians demonstrated significantly greater cortical thickness in the bilateral superior frontal gyrus and the right middle frontal gyrus compared to non-musicians. These findings are consistent with prior research suggesting that increased cortical thickness in the superior frontal gyrus may reflect the substantial cognitive demands placed on networks involved in mnemonic retention, monitoring, and retrieval during many years of musical training (Bermudez et al., 2009; Petrides and Pandya, 2002). Similar patterns were also observed in child musicians (ages 9–11) and elderly musicians (ages 50–80), where the posterior segment of the superior frontal gyrus was thicker than in non-musicians (Ghosh et al., 2024; Habibi et al., 2020), suggesting that frontal regions play an important role in lifetime musical development.

Additionally, musicians showed greater cortical thickness in the caudal and rostral middle frontal gyrus, consistent with previous studies (Bermudez et al., 2009). This region has been shown to activate during both passive listening and explicit sound identity comparison tasks, suggesting its involvement in developing a stable representation of the environment in the face of variable auditory information – an essential function for musicians (Giordano et al., 2014). Furthermore, the precentral gyrus, a core sensorimotor area, was also thicker in musicians, as consistent with prior research (Bermudez et al., 2009). Long-term training with drums and wind instruments has been associated with plasticity in the region, and is linked to motor execution (Bruchhage et al., 2020; Choi et al., 2018). In addition, clinical and behavioral studies have shown that this cluster is further associated with motor processes requiring perceptive feedback and strong attentional control (Bruchhage et al., 2020), suggesting that musical training may enhance some aspects of attention, even in an extra-musical context (Roman-Caballero et al., 2021).

Altogether, these findings provide structural evidence for musicians’ cognitive advantages in frontal regions of the brain and perceptual processing strengths in the precentral gyrus. Notably, both regions are also key components of the functional brain networks that are activated when listening to music (Liu et al., 2021, 2022). Future studies may benefit from incorporating these regions into structural network analyses in order to further explore the influence of music on brain plasticity.

### 4.2 Cortical thickness—interactions between group and gender

When examining the interactions between musical training and gender, the left bankssts was found to be thicker in male musicians compared to male non-musicians. Bankssts thickness is an important brain index for gender differences in brain development and executive function, including working memory, reading comprehension, and fluency (Wierenga et al., 2019). In Wierenga’s two-year longitudinal study, it was found that the left bankssts in boys had a steeper decline in surface area compared to the left bankssts in girls, suggesting a distinct developmental trajectory. Given the prominent executive functions of musicians, the thicker bankssts in male musicians may reflect a developmental advantage.

Interestingly, however, the left bankssts was found to be the area of the brain with the highest *β*-amyloid deposition in cognitively normal elderly adults (Guo et al., 2020), making it a potential early biomarker for Alzheimer’s disease (AD) (Wee et al., 2013). The thicker bankssts in male musicians compared with male-nonmusicians suggests that musical training and cortical thickness may serve as analytical evidence for cognitive processing and may even be used to predict cognitive decline, ultimately enhancing clinical diagnostic strategies.

### 4.3 Fractal dimensionality and sulcal depth—musicians versus nonmusicians

Our analysis revealed that musicians exhibited higher fractal dimensionality and sulcal depth in the left fusiform and right postcentral gyri compared to non-musicians. Fractal dimensionality is a useful measure of cortical complexity, ranging from molecular architecture to whole-brain morphometry (Di Ieva et al., 2014, 2015; Meregalli et al., 2022). Musical training has been associated with increased activation in the left fusiform gyrus (Schmithorst and Holland, 2003), which has also been implicated in the perception of sound richness in a PET study (Satoh et al., 2015). Furthermore, in a systematic review of the literature on clinical and nonclinical samples, the fractal dimensionality of the left fusiform was also found to be higher in healthy participants compared to patients with bipolar disorder (Meregalli et al., 2022), suggesting that this structural advantage may reflect enhanced emotional regulation and cognitive development in musicians.

Sulcal depth has been studied as an important neuroimaging biomarker for brain diseases and has been widely used to study the morphological characteristics of cerebral folding (Im et al., 2008; Shin et al., 2022). The increased sulcal depth in the fusiform gyrus seen in musicians may reflect domain-specific perceptual expertise. This region’s close relationship with the postcentral gyrus further supports its role in cognitive development (Yao et al., 2023). While fractal dimensionality and sulcal depth have not been discussed in conjunction with existing research, the integration of these two metrics may lead to improved computational analyses of brain structure.

### 4.4 Fractal dimensionality—interactions between group and gender

When considering the interaction between musical training and gender, female musicians showed greater fractal dimensionality in the left fusiform gyrus compared to female non-musicians, while male musicians showed greater fractal dimensionality in the right postcentral gyrus compared to male non-musicians. These gender-specific patterns may suggest that female musicians are more attenuated to emotional perception via fusiform activation, while male musicians may engage sensorimotor processes via the postcentral gyrus (Shah et al., 2021).

### 4.5 Gyrification—musicians versus non-musicians

In terms of gyrification, non-musicians exhibited greater cortical folding in several regions, including the bilateral insula, right inferior and superior parietal lobules, posterior cingulate cortex, and superior temporal gyrus compared to musicians. Gyrification is often quantified using the ‘gyrification index’ (Zilles et al., 1988), and higher values indicate a higher degree of cortical folding, a developmental marker for brain maturation (White et al., 2010). In contrast to cortical thickness, fractal dimensionality, and sulcal depth—gyrification was more prominent in non-musicians. These differences were widely distributed across multiple brain regions, including the insula, parietal lobules, central gyrus, and temporal gyrus. Cortical folding is the result of complex cellular and mechanical processes that involve neural stem progenitor cells and their lineages, the migration and differentiation of neurons, and the genetic programs that regulate and fine-tune these processes (Fernandez and Borrell, 2023). Increased gyrification has been seen in the auditory cortices of both young and elderly musicians (Benner et al., 2017; Rus-Oswald et al., 2022). The occurrence of nonmusicians’ increased gyrification here may be due to a false outcome caused by insufficient sample size. In the future, an increased sample size will allow for the investigation of differences in gyrification between musicians and nonmusicians.

### 4.6 Limitations

The current study had several limitations. First, the relatively small sample size in musicians and non-musicians necessitates future follow-up studies with larger samples to validate these findings. Second, although singing musicians and instrumental musicians have similar or overlapping neural functions or structures, there are also differences between them (Ghosh et al., 2024); for example, singers may exhibit enhanced activation in language-dominant brain regions more than instrumentalists; this difference stems from the unique nature of vocal motor training, which involves the physical production of sound through speech-like movements, contrasting with playing an instrument using one’s hands (Christiner and Reiterer, 2015). Therefore, it is necessary for future research to further group instrumental musicians and singing musicians for more detailed investigation. Third, no behavioral measure was utilized in the current study, which may not adequately capture the multifaceted aspects of music acquisition. Future studies should therefore incorporate behavioral measures and explore their associations with brain structural brain morphometry changes to achieve a more integrative understanding.

## 5 Conclusion

As one of the few studies to systematically analyze the effects of musical training on structural brain development, the current study identified key differences in brain morphology between musicians and non-musicians across four measures: cortical thickness, fractal dimensionality, gyrification, and sulcal depth. Our findings suggest that increased cortical thickness in the frontal lobe, along with greater fractal dimensionality and sulcal depth in the left fusiform and right postcentral gyri, provide important structural evidence for enhanced brain development in musicians. Conversely, stronger gyrifications in multiple brain regions in non-musicians offers new insight into the development of brain plasticity.

Despite these findings, our current understanding of the brain’s structural adaptations to music is still limited. Rather than relying on single morphological indicators, future research should aim to develop an analytical method that integrates multiple morphological indicators in order to provide a more comprehensive understanding of how the brain processes music and how musical training affects neuroplasticity.

## Data Availability

The original contributions presented in the study are included in the article/supplementary material, further inquiries can be directed to the corresponding author.

## Footnotes

^1^ https://openneuro.org/datasets/ds003146/versions/1.0.2
